# Analysis of heart rate variability and subtle ECG changes based on machine learning for objective assessment of the psychological state of military personnel

**DOI:** 10.3389/fpsyg.2026.1688230

**Published:** 2026-02-27

**Authors:** Illya Chaikovsky, Ivan Senko, Mykola Budnyk, Viktor Matsyshyn, Tetiana Ryzhenko, Vitaliy Budnyk, Oleksandr Romanchuk, Anton Popov, Petro Stetsyuk

**Affiliations:** 1 Department of Contactless Control Systems, V.M. Glushkov Institute of Cybernetics of the National Academy of Sciences of Ukraine, Kyiv, Ukraine; 2 Department of State Administrative, State Institution of Science «Center of Innovative Healthcare Technologies», Kyiv, Ukraine; 3 Department of Internal and Family Medicine, Lesya Ukrainka Volyn National University, Lutsk, Ukraine; 4 Department of Therapy and Rehabilitation, Ivan Bobersky Lviv State University of Physical Culture, Lviv, Ukraine; 5 Department of Electronic Engineering, Igor Sikorsky Kyiv Polytechnic Institute, Kyiv, Ukraine; 6 Faculty of Applied Sciences, Ukrainian Catholic University, Lviv, Ukraine

**Keywords:** correlation analysis, ECG, heart rate variability, machine learning, military personnel, psychological questionnaires, signal analysis

## Abstract

**Introduction:**

The implementation of objective methods for rapid assessment of the psychological and physiological readiness of military personnel is an extremely relevant task. The cardiovascular system acts as a “mirror” of functional and psychological state. The most common and accessible method for the objective study of the cardiovascular system remains electrocardiography (ECG). This study aims to develop a technology for objective monitoring of the psycho-emotional state and overall functional condition of personnel in the Ukrainian Defense Forces using miniature ECG devices and in-depth analysis of ECG signals with artificial intelligence.

**Methods:**

Using an innovative ECG device, 90 servicemen, average age of 38 years, undergoing sanatorium treatment and rehabilitation at the Central Military Clinical Sanatorium “Khmilnyk” were examined. The examination was conducted on the first or second day after the start of sanatorium treatment. ECG and HRV analysis were performed using our previously developed Universal Scoring System. The results of ECG analysis from limb leads in 6 leads were compared with 4 well-known psychological self-assessment methods: Beck Anxiety Scale, PCL-5, PHQ-9, as well as a formalized psychologist’s conclusion. Correlation analysis and Sequential Feature Selector were used.

**Results:**

Forty ECG/HRV features were selected for each of the four psychological methods to find the maximum *R*^2^ metric. The highest number of reliable correlations between ECG and HRV parameters and psychological tests was found for the Beck Anxiety Scale. The same can be said when using feature selection via machine learning. The cross-validated *R*^2^ scores for the training and test sets in the case of the Beck Anxiety Scale were 0.520/0.359, respectively. Similar results were obtained for the Preliminary Psychological.

**Conclusion:**

The study’s results demonstrate the potential for significant prediction of routine psychological assessment outcomes based on in-depth analysis of ECG and HRV, especially regarding the Beck Anxiety Scale and Preliminary Psychological Conclusion.

## 1 Introduction

The presence of Ukraine in a state of large-scale war, with all its negative social consequences, has led to a deterioration in the psycho-emotional state of various segments of the population and the emergence of psychogenic disorders. The intensity of several conditions, which can be interpreted as pre-depressive, is significantly increasing. Against the backdrop of these events, professional psychological diagnostics are an extremely important task. This will not only allow for timely, accurate diagnosis but also appropriate treatment and therapy. Another important task is to assess the effectiveness of therapeutic and rehabilitative measures in patients with psycho-emotional disorders.

The primary tool for addressing this issue is a psychometric assessment based on self-reporting, which is widely used in both academic and non-academic settings, usually in the form of a questionnaire. However, the quality of data obtained through self-reporting largely depends on respondents’ ability and willingness to provide accurate answers. When respondents are interested and focused, they are more likely to consider the questions and express their true thoughts carefully. Conversely, when respondents are unmotivated, irritated, or tired, they are more likely to give nonsensical or even random answers (Oppenheimer et al., 2009), leading to measurement errors. This is most often the case in conditions of large-scale war.

Therefore, the implementation of objective methods for rapid assessment of soldiers’ psychological and physiological readiness is a highly relevant task. A soldier’s psychological and physiological readiness collectively constitute their functional state (FS). In general, FS requires an integrated assessment of functions that directly or indirectly determine the effective performance of soldiers in specific conditions of the activities of different types and branches of the military (forces).

The general components of functional state are recognized as cognitive, psycho-emotional, and resource-energy. The cognitive component characterizes the ability to process information and is assessed using blank tests. The psycho-emotional component characterizes the emotional state, significantly influences the subconscious, and is primarily evaluated through subjective questionnaires, as well as in a diagnostic interview with a psychologist. Another modern tool for objective analysis of the psycho-emotional component is several relatively new hardware methods, such as the assessment of specific spectral parameters of heart rate variability (HRV).

Accumulating evidence highlights heart rate variability (HRV) as a sensitive biomarker of autonomic dysregulation associated with psychological stress, anxiety, depression, and trauma-related disorders. Recent meta-analyses demonstrate a consistent reduction in high-frequency (HF) power, root-mean-square of successive differences (RMSSD), and overall HRV in individuals exposed to acute or chronic stressors, reflecting impaired vagal modulation and increased sympathetic drive, including respiratory power (Chalmers et al., 2014; Kim et al., 2018; Shaffer and Meehan, 2020; Romanchuk, 2024). Large cohort studies performed between 2020 and 2023 further confirm that lower resting HRV is associated with reduced psychological resilience, poorer emotional regulation, and greater vulnerability to stress-related psychopathology (Souza et al., 2013).

In populations with trauma exposure, including military personnel and war-affected civilians, HRV indices have been shown to correlate with symptom severity of post-traumatic stress disorder (PTSD), hyperarousal, and dysregulated stress responses (D’Souza et al., 2019). Importantly, HRV has also emerged as a predictor of treatment response in psychotherapy and mind–body interventions, indicating its utility not only for risk assessment but also for monitoring recovery trajectories (D’Souza et al., 2019; Immanuel et al., 2023). Taken together, contemporary evidence supports the integration of HRV analysis as an objective, non-invasive tool for evaluating psychophysiological stress load and autonomic imbalance in individuals affected by war-related psychological trauma.

The resource-energy component describes the body’s ability to provide metabolic resources for performing specific activities and is evaluated using objective physiological methods.

The cardiovascular system plays the role of a “mirror” of FS, especially in the context of its resource-energy component. The most common and accessible method of objective examination of the cardiovascular system remains electrocardiography (ECG) and its derivative HRV. In recent years, the well-known ECG method has undergone further significant development, primarily involving the development of methods for analyzing ECG signals using artificial intelligence, as well as the miniaturization of electrocardiographs. In recent years, the V.M. Glushkov Institute of Cybernetics of the National Academy of Sciences of Ukraine has developed several versions of an innovative portable software-hardware ECG system designed, among other things, for use in the units of the Ukrainian Defense Forces.

The goal of this study is to develop technology for the objective monitoring of the psycho-emotional state and, in general, the functional state of personnel in the Defense Forces of Ukraine, utilizing miniature ECG devices and in-depth analysis of the ECG/HRV signal.

## 2 Materials and methods

### 2.1 Study subjects

Using an innovative ECG device (Bocharov et al., 2023), 90 military personnel, average age of 38 years, were examined. They were undergoing sanatorium treatment and rehabilitation at the Central Military Clinical Sanatorium “Khmilnyk” due to musculoskeletal diseases of mild or moderate severity. All servicemen had previously been in a combat zone for at least 3 months. The exclusion criteria were as follows: 1. unable or unwilling to cooperate according to the study protocol; 2. malignant hypertension (= sitting SBP > 180 mmHg and/or sitting DBP > 105 mmHg); 3. compound rhythm disturbances (frequent PVC, paroxysmal SVT/VT, atrial fibrillation); 4. active wasting disease including cancer; and 5. severe psychological and/or emotional problems which would render the informed consent invalid.

**FIGURE 1:**
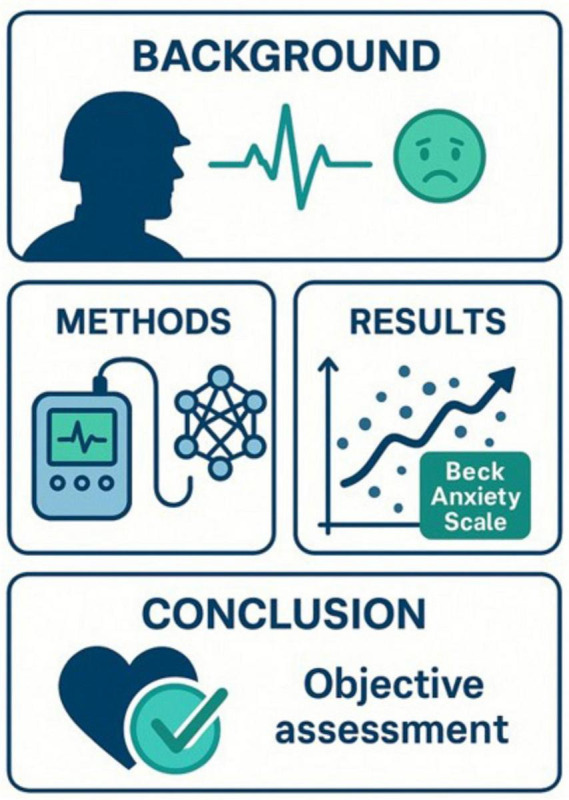
Graphical abstract.

The examination was conducted on the first or second day after the start of sanatorium treatment. During routine ECG analysis, no significant deviations were detected in any of the examined individuals, indicating no substantial changes in the ECG according to the Minnesota coding. An objective assessment of their condition was conducted based on an in-depth analysis of minor ECG changes and HRV, using the methodology described in Chaikovsky’s work (2020) (Colan, 2013; Chaikovsky, 2020). The results of the limb ECG analysis in 6 leads were compared with 4 well-known assessments (target variables) routinely used in practical psychology, specifically, 3 self-assessment methods: the Beck Anxiety Scale (Beck et al., 1988), the PTSD checklist for DSM-5 (PCL-5) (Forkus et al., 2023), the PHQ-9 questionnaire for depression severity (Kroenke et al., 2001), as well as a formalized psychologist’s conclusion in the form of a scale from 1 (normal psycho-emotional state) to 4 (significant psycho-emotional disturbances), which was created based on a semi-standardized individual interview (Figure 1).

### 2.2 Methods

**FIGURE 2:**
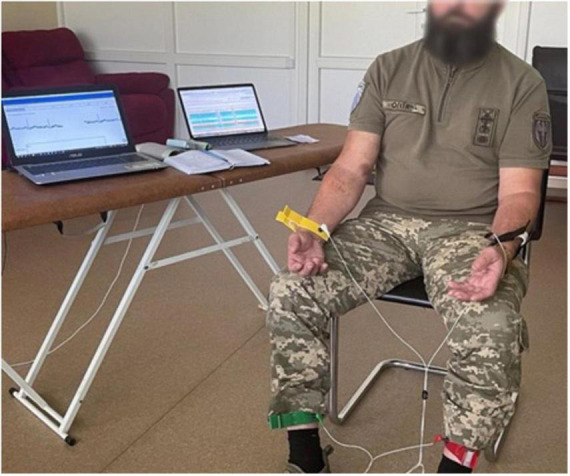
Examination of military man using innovative ultra-compact ECG device and dedicated software.

The examination was conducted using the fully certified “Cardio + P” innovative device, developed by V.M. Glushkov Institute of Cybernetics of the National Academy of Sciences and manufactured by “Metekol” LLC. This system consists of an ultra-compact ECG device and dedicated software (Figure 2). The software provides multi-faceted analysis of ECG and HRV using a method (Chaikovsky, 2020) of ECG scaling that we developed, capable of providing a quantitative assessment of the subtle changes in the ECG signal. The idea of our approach is, firstly, to measure the maximum number of routine and original parameters of ECG and HRV. Secondly, to position each parameter on a scale between the absolute norm and extreme pathology.

A problem arises when attempting to convert these parameters into a compact and transparent form suitable for decision-making—that is, by applying the so-called “dimensionless” form.

The starting point of this method is the mean value of each parameter *within* the normal range.

Next, the range of normal values—representing the quantified limits of the organism’s functioning—must be established, which serves as the standard. As a rule, the boundaries of the normal range are the values *one standard deviation away* from the mean value of the parameter, i.e., M ± σ.

A hybrid approach is used to obtain these parameters: the values for the most well-known, basic ECG parameters, such as the amplitude and duration of the main waves and ECG intervals, were taken in the most authoritative source in this field (Macfarlane et al., 2010).

The remaining parameters were obtained *by* processing a database of healthy volunteers.

This database consists of 1,112 electrocardiograms collected within our own research (881 male subjects, mean age 28 ± 5.2 years).

This group consisted of healthy volunteers with no complaints, no history of any chronic diseases, and normal results of routine tests. Most of the members of this group were cadets and teachers of higher military academies.

In the next step, a procedure establishes linear relationships between the discrete values in absolute units and the number of points that correspond to that discrete value. As a result, for each individual indicator of the first level of analysis, a linear scale of correspondence between the absolute values and the number of points was obtained based on piecewise linear interpolation. It is obvious that individual ECG and HRV indicators reflect only partial aspects of the phenomenon under study; in addition, they can be multidirectional. Therefore, to draw a *definitive* conclusion, a generalizing index is needed that synthesizes the effects of the individual components (OECD, 2008).

Therefore, the software is built on a hierarchical principle and consists of four levels:

1. The lower level includes a set of individual indicators that describe:

(a) various aspects of the ECG;

(b) amplitude-time indicators and waveforms of the ECG;

(c) the presence of main disturbances in frequency, rhythm, and sequence of heart muscle contractions (in other words, disturbances of heart rhythm).

1. The second level consists of groups of related indicators that have a close physiological significance.
2. The third level is represented by three integral blocks, each reflecting different aspects of the functioning of the cardiovascular system, which can be assessed using ECG and HRV. These blocks are for evaluating regulation, myocardial condition, diagnosis of arrhythmias, as well as psycho-emotional status. The development of the proprietary composite index for assessing psychoemotional state is based primarily on the work of Mccraty and Shaffer (2015).
3. The fourth, highest level, is a general integral indicator of the cardiovascular system.

The composite index is determined by averaging the values of the individual parameters *that constitute it*. The integral value of the higher-level composite index is formed by averaging the integral values of the “lower” composite indices. The final results *are* also presented within the 100-point interval scale. The construction of composite indices is based on a database consisting only of healthy volunteers. The appropriate statistical analysis to apply in this case is one-sample *statistics*. It is most natural to investigate various features of the distribution of the parameters under study, with the aim of proving the stability of statistical conclusions. The Huber robustness test was chosen to assess the stability of the composite *indices* (Huber, 1981). Only those indices that passed this test were included in the statistical analysis. In addition, the HDB Scan method was used to cluster each of the robust composite indices. It was shown that in a large group of healthy volunteers, each index formed a generally homogeneous, dense cloud of points.

The proposed scaling method was developed specifically to address practical problems and is already widely used in Ukraine and abroad to solve a variety of different issues.

The mathematical processing of the data array consisted of the following stages:

1. Correlation analysis. The advantages of correlation analysis are that it considers each feature separately for each target variable, allowing the identification of statistical relationships between all features and variables. For the analysis, two statistical correlation criteria will be used—Pearson and Spearman.
2. Selection of informative features from the initial set of parameters based on machine learning (ML) with a linear regression as a core method. For machine learning and statistical processing, Python version 3.11 was used, along with data processing, scientific computing, and ML libraries: Pandas version 2.2 (McKinney, 2010), SciPy version 1.14 (Virtanen et al., 2020), and Scikit-learn version 1.6 (Pedregosa et al., 2011). Functions *scipy.stats.pearsonr* and *scipy.stats.spearmanr*, taken from SciPy, were used for correlation analysis, as well as the statistical test *scipy.stats.wilcoxon*.

**FIGURE 3:**
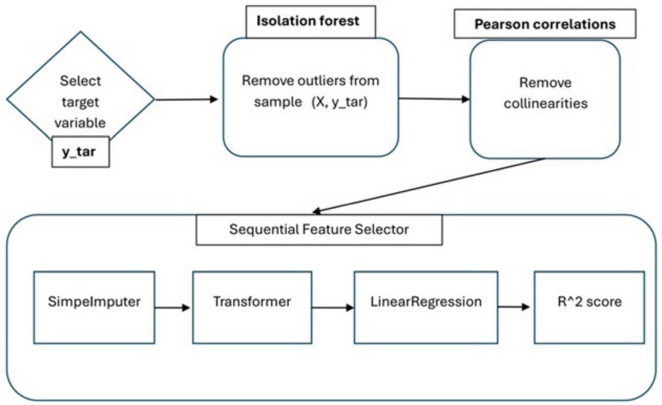
Block diagram of the feature selection algorithm based on ML.

The overall flowchart of the feature selection algorithm is shown in Figure 3.

Linear regression is chosen as a core ML model, because of its interpretability for the feature influence. Sequential feature selector runs step-wise forward from 1 to 40 features for each of the four psychological target variables. The number of the selected predictors corresponds to the maximum of cross-validated *R*^2^ metric calculated on each step of increasing the number of predictor variables.

The collinear features are removed using Pearson correlation with the threshold 0.7 after the outlier removal. The number of removed outlier observations for the each of the combination (target variable, predictors) are shown in Table 1. The Python classes used for the aforementioned statistical methods are listed in Table 2.

**TABLE 1:** Detected and removed outliers for each target variable.

Target variable	PTSD checklist—Military Version (PCL-5)	Patient Health Questionnaire-9 (PHQ-9)	Beck Anxiety Scale	Preliminary Psychological Conclusion
Total patients	90	90	90	90
Outliers detected	6	7	8	6
Processed records	84	83	82	84

**TABLE 2:** Python classes for the feature selection algorithm.

Method	Python class
Linear regression	Sklearn.linear_model.LinearRegression
Outlier detection	Sklearn.ensemble.IsolationForest
Missing value imputation	Sklearn.impute.SimpleImputer
Correlation analysis	Pandas.DataFrame.corr(method = “Pearson”) Pandas.DataFrame.corr(method = “spearman”)
Feature transformation	Sklearn.preprocessing.quantiletransformer Sklearn.preprocessing.PowerTransformer
Scoring metric	LinearRegression.score
Feature selection	Sklearn.feature_selection.SequentialFeatureSelector

#### 2.2.1 Feature selection procedure and control of overfitting

To ensure a reproducible evaluation and to avoid information leakage in a limited-sample setting, we organized the analysis as a training-only procedure followed by evaluation on a held-out test set (Table 7). Feature selection was performed on the training set using a stepwise forward sequential feature selection procedure with ordinary least squares linear regression as the base estimator. The procedure starts from an empty feature set and iteratively adds one feature at a time. At each step, the candidate feature that yields the largest improvement in mean cross-validated R-squared (using five-fold cross-validation) is added. This process was repeated up to a maximum of forty candidate features, and the final number of selected features was chosen as the one that provided the best average cross-validated performance on the training set. This approach selects model complexity empirically rather than fixing the number of predictors in advance, which is recommended when the number of candidate features is large compared with the number of observations (Guyon and Elisseeff, 2003; James et al., 2023).

Several complementary strategies were employed to control overfitting and improve generalization. First, we reduced collinearity by removing highly correlated predictors (Pearson absolute correlation > 0.7), because redundant predictors can inflate variance and lead to unstable coefficient estimates. Second, the outlier handling described in Table 1 was applied using only information from the training data within each modeling run to avoid optimistic bias. Third, all preprocessing steps—including the selected feature transformation or scaling option, outlier handling, correlation-based filtering, and feature selection—were carried out within the cross-validation workflow, so that each validation fold remained completely unseen during fitting and selection. This is consistent with established guidance that feature selection must be nested within resampling to avoid selection-induced bias and overly optimistic performance estimates (Cawley and Talbot, 2010). Finally, we report both cross-validated performance on the training set and performance on an independent held-out test set (Table 7).

The test set was not used at any stage of preprocessing, feature selection, or model fitting, providing an external check against overfitting.

#### 2.2.2 Hyperparameter tuning and evaluation of alternative algorithms

Hyperparameter tuning was intentionally limited in this study to reduce model-selection flexibility on a small dataset. The primary predictive model was ordinary least squares linear regression, which does not involve regularization hyperparameters. With respect to preprocessing, we compared several standard feature transformation and scaling options (Table 7) and selected the configuration that maximized the average five-fold cross-validated R-squared on the training set. No additional automated hyperparameter optimization procedure, such as grid search or random search, was performed (Bergstra and Bengio, 2012).

We selected linear regression as the main model because interpretability of the direction and magnitude of ECG and HRV feature effects was a core study objective, and linear coefficients provide a transparent link between physiological markers and psychological scores (James et al., 2023). Alternative machine learning algorithms were not exhaustively benchmarked in the present work; this was a deliberate design choice to prioritize interpretability and to avoid overly optimistic conclusions that can arise from extensive model searching in a limited-sample setting. Future work on larger cohorts will include systematic comparisons with regularized linear models (Ridge, Lasso, and Elastic Net) and non-linear regressors (for example, kernel-based methods and tree-based ensembles) using nested cross-validation and explicit hyperparameter optimization to quantify any potential accuracy gains while controlling generalization error (Hastie et al., 2009; Cawley and Talbot, 2010; Bergstra and Bengio, 2012).

The study was conducted in accordance with the Declaration of Helsinki and approved by the Ethics Committee of Glushkov Institute of Cybernetics of the National Academy of Sciences of Ukraine (Kyiv) and ethics committee of Clinical Sanatorium “Khmilnyk” (Protocol No.2 dated March 15, 2019). Written informed consent was obtained from all participants, and their personal data were anonymized to ensure confidentiality.

## 3 Results

### 3.1 Correlation analysis

As previously noted, observations with missing data (i.e., absent recorded feature values) are discarded prior to processing. First, we calculate Pearson’s r-correlations, then sort the features within the target groups by their significance levels. Suppose we use a significance level of *p* < 0.01 for the correlation. In that case, we obtain 2 correlated features for the PTSD Checklist—Military Version (PCL-5), 8 correlated features for the Patient Health Questionnaire-9 (PHQ-9), 26 correlated features for the Beck Anxiety Scale, and 3 correlated features for the Preliminary Psychological Conclusion. Table 3 shows only the features with a significance level of *p* < 0.05, which are present in only two psychological tests: PHQ-9 and Beck Anxiety Scale.

**TABLE 3:** Statistically significant (*p* < 0.01) Pearson correlations between ECG/HRV parameters and psychological tests.

No	ECG/HRV parameter	Correlations coefficient	Significance
**Patient health questionnaire-9 (PHQ-9)**			
1	Psycho-emotional status (composite index)	−0.271	0.00967
2	McCraty psycho−emotional index	−0.271	0.00967
3	Mashin psycho-emotional index	0.243	0.0210
4	Amplitude S-wave (μV) (lead AvL)	−0.237	0.0243
**Beck anxiety scale**			
1	Amplitude Q-wave (μV) (lead II)	−0.388	0.000158
2	Amplitude S-wave (μV) (lead AvR)	−0.358	0.000537
3	Amplitude Q-wave (μV) (lead AvR)	0.319	0.00221
4	Amplitude Q/R ratio (lead AvR)	−0.442	0.00340
5	Amplitude Q/R ratio (lead II)	0.301	0.00390
6	McCraty psycho-emotional index	−0.300	0.00413
7	Psycho-emotional status (composite index)	−0.300	0.00413
8	Mashin psycho-emotional index	0.293	0.00512
9	Amplitude ratio Q/R (lead I)	0.236	0.0257
10	Amplitude Q (μV) (lead AvF)	−0.226	0.0322
11	Duration PQ (sec)	0.222	0.0354
12	Amplitude Q (μV) (lead I)	−0.217	0.0396
13	Amplitude ratio R/P (lead II)	0.211	0.0460
14	Myocardial reserves status (composite index)	0.209	0.0480

Then Spearman’s ρ-correlations have been calculated. At a significance level of *p* < 0.01, 4 significant features were obtained for the PCL-5, 11 significant features for the PHQ-9, 20 significant features for the Beck Anxiety Scale, and 9 correlated features for the Preliminary Psychological Conclusion. Similar to Pearson correlations, Table 4 presents ECG/HRV parameters according to the *p* < 0.05 condition, which is present for all four psychological tests in this case.

**TABLE 4:** Statistically significant (*p* < 0.01) spearman correlations between ECG/HRV parameters and psychological tests.

No	ECG/HRV parameter	Correlations coefficient	Significance
**PTSD checklist—military version (PCL-5)**			
1.	Q/R amplitude ratio (lead I)	0.236	0.0259
2.	R-wave amplitude (μV) (lead AvR)	0.229	0.0301
3.	ECG interval duration index	0.224	0.0336
4.	Q-wave amplitude (μV) (lead I)	−0.216	0.0405
5.	Q-wave duration (sec)	0.215	0.0415
6.	R/T amplitude ratio (lead AvR)	−0.204	0.0435
**Beck anxiety scale**			
1.	Amplitude ratio Q/R (lead AvR)	−0.516	0.000474
2.	Amplitude S-wave (μV) (lead AvR)	−0.346	0.000830
3.	Amplitude Q-wave (μV) (lead AvR)	0.296	0.00460
4.	Amplitude Q-wave (μV) (lead II)	−0.272	0.00939
5.	AvR lead score	−0.271	0.00970
6.	Sylvester score	−0.271	0.00970
7.	CIIS score	−0.271	0.00970
8.	Amplitude Q-wave (μV) (lead I)	−0.251	0.0168
9.	Amplitude ratio Q/R (lead II)	0.249	0.0179
10.	Amplitude ratio Q/R (lead I)	0.247	0.0196
11.	Q duration (sec)	0.229	0.0302
12.	Psycho-emotional status (composite index)	−0.227	0.0312
13.	McCraty psycho-emotional index	−0.227	0.0312
14.	Area ratio P/QRS (lead I)	−0.228	0.0398
15.	Amplitude S-wave (μV) (lead III)	−0.208	0.0495
**Preliminary psychological conclusion**			
1.	PQ duration (sec)	0.232	0.0287
2.	Area ratio P/QRS (lead I)	0.215	0.0433

Thus, it is possible to summarize the total number of significant features based on Pearson’s correlation (Table 5) and Spearman’s correlation (Table 6).

**TABLE 5:** Summary for Pearson’s correlations.

Significance level for r-correlations (Pearson)	PCL-5	PHQ-9	Beck anxiety scale	Preliminary psychological conclusion
*p* < 0.001	0	0	2	0
*p* < 0.01	0	1	6	0
*p* < 0.05	0	2	7	0
*p* < 0.1	2	4	10	3

**TABLE 6:** Summary for Spearman’s correlations.

Significance level for r-correlations (Spearman)	PCL-5	PHQ-9	Beck anxiety scale	Preliminary psychological conclusion
*p* < 0.001	0	0	2	0
*p* < 0.01	0	0	3	0
*p* < 0.05	1	5	8	3
*p* < 0.1	3	6	4	6

### 3.2 Feature selection based on machine learning models

Applying the submission algorithm shown in Figure 1, we obtain the results presented in Table 7. The results with the highest scores for the training tests are listed, along with results obtained using different transformers when their scores are close. At the same time, preference is given to models with fewer features to reduce the risk of overfitting. On the other hand, all tests were validated using five-fold cross-validation to ensure that they are robust on the observed, although small, dataset.

**TABLE 7:** Scores of the educational test and feature transformers.

Target variable	Number of selected features	Train score	Test score	Feature transformer	Target transformer
PCL-5	30	0.369	0.294	Quantile	None
	34	0.370	0.293	Quantile	Box-Cox
PHQ-9	30	0.311	0.248	Quantile	Box-Cox
	33	0.341	0.238	Quantile	
	30	0.342	0.236	Quantile	Box-Cox
Beck anxiety scale	26	0.520	0.359	Quantile	Yeo-Johnson
Preliminary psychological conclusion	27	0.517	0.268	Quantile	Box-Cox

The features selected using the provided algorithm are listed in Table 8. Only the top 10 features with the highest scores are shown here. The total number of selected features is given in Table 7. It is clear that as the number of features increases, their significance decreases.

**TABLE 8:** The most informative ECG/HRV features for each psychological test, selected using machine learning.

No	PCL-5	PHQ-9	Beck anxiety scale	Preliminary psychological conclusion
1	ECG interval duration index	HRV composite index	Amplitude Q-wave (μV) (lead AvR)	HF-SVD QRST analysis (composite index)
2	PNN50, %	PNN50, %	ECG interval duration index	ECG waves amplitudes index (lead II) (composite index)
3	HF	QTcF duration (sec)	R/P amplitude ratio (lead II)	Integral reserves status (composite index)
4	SDSD, ms	Lead I score	P-wave area (μV*sec) (lead I)	Myocardial reserves status (composite index)
5	Myocardial reserves status (composite index)	Myocardial reserves status (composite index)	Amplitude P-wave (μV) (lead AvR)	P-wave area (μV*sec) (lead I)
6	Risk of the occurrence of significant cardiovascular events (composite index)	J-point dislocation (μV) (lead I)	PNN50,%	ECG waves amplitudes index (lead AvF) (composite index)
7	Duration of P-wave (sec)	Composite index of functional state	HF	T-wave amplitude (μV) (lead I)
8	Activity of subcortical levels of regulation (HRV composite index)	Psycho-emotional status (composite index)	Mashin psycho-emotional index	QTcF duration (sec)
9	ECG waves amplitudes index (lead III) (composite index)	Macruz index P/(PQ-P)	Amplitude Q-wave (μV) (lead II)	Lead AvR score
10	QRS area (μV*sec) (lead I)	Detrended Fluctuation Analysis (DFA) of HRV	Amplitude S-wave (μV) (lead III)	Activity of subcortical levels of regulation (HRV composite index)

## 4 Discussion

This work once again raises the question of the relationship between objective and subjective methods of psychodiagnostics. Objective methods of psychodiagnostics are those in which the results are minimally dependent on the diagnostician and are based on quantitative, formalized criteria. These are primarily instrumental methods. Subjective methods (questionnaires, interviews, projective tests) involve self-assessment or interpretation, where the attitudes of the subject or psychologist have a greater influence. Objective methods provide reliable data, while subjective methods provide depth of understanding and context, creating a holistic picture of the personality within the framework of a comprehensive diagnosis. Naturally, these two types of diagnostics complement each other. At the same time, psychodiagnostics in field and similar conditions requires the adaptation of standard methods to unpredictable, often extreme, situations. Here, the most important factors are the efficiency, reliability, and practicality of the tools used, with a focus on behavioral prediction. Consequently, the potential value of instrumental methods in this context increases.

The results of our study demonstrate the potential for significant prediction of the outcomes of routine psychological assessments of patients based on an in-depth analysis of ECG and HRV within the framework of the developed scoring system (Zharinova et al., 2015). Undoubtedly, the greatest number of highly reliable links between ECG and HRV parameters and psychological tests were found for the Beck Anxiety Scale. The same can be said when using feature selection through machine learning—the cross-validated *R*^2^ test score ensures that the identified influencing variables represent true dependencies between ECG/HRV characteristics and psychological indicators, rather than random or noise-related dependencies (Sen’ko, 2013). The prediction model was chosen as linear regression due to the high interpretability what correspond our goal of detection of the variable influence to the target indicators. In addition, coefficients of linear models allow adjustments under the conditions of additional information being available (Sen’ko, 2013; Sen’ko, 2014).

The comparison of the forecast for the training and validation (test) groups confirms the effectiveness of the applied algorithm for selecting informative features. The prediction accuracy of the relationship for the validation sample for all four psychological methods was lower than for the test sample, which aligns with theoretical expectations. The most consistent results correspond to other findings for the Beck Anxiety Scale. According to Pearson or Spearman correlation analysis, 15 features were also selected (Tables 3, 4), and the training/validation metric values are approximately 0.52/0.35, respectively, as shown in Table 7.

The Beck Anxiety Scale is a well-known classic psychological test, characterized by the fact that 15 out of 21 items on this scale measure physiological symptoms. Therefore, it functions more accurately in cases of anxiety disorders with a high somatic component. Such a profile of disorders was observed in the group we studied.

The relationship between HRV indicators and psycho-emotional state is well known. HRV is a measure that reflects changes in the intervals between heartbeats and serves as a marker of autonomic nervous system (ANS) activity. Since the ANS plays a key role in regulating emotional and stress responses, HRV is widely used to assess psycho-emotional health. Psychological states such as stress, anxiety, or depression affect the balance between the sympathetic (activates the “fight or flight” response) and parasympathetic (promotes relaxation and recovery) branches of the ANS. High HRV typically indicates dominance of parasympathetic activity, associated with a relaxed state, while low HRV indicates dominance of the sympathetic system, which is characteristic of stress or emotional tension. Therefore, HRV is a promising non-invasive tool for monitoring psycho-emotional health.

For analyzing the psycho-emotional state using HRV, various parameters are used: time-domain parameters such as SDNN (standard deviation of RR intervals) and RMSSD (root mean square of successive differences), as well as frequency-domain parameters like LF (low-frequency component), HF (high-frequency component), and the LF/HF ratio. For example, a decrease in HF, which reflects parasympathetic activity, and an increase in the LF/HF ratio can indicate a high level of stress or anxiety. Studies confirm that people with depression or anxiety often have reduced HRV, which correlates with their emotional state. Additionally, HRV helps evaluate the effectiveness of therapies such as meditation or psychotherapy, making it a valuable biomarker in clinical practice and scientific research, particularly when assessing the psycho-emotional state of military personnel (Matsyshyn et al., 2023).

However, HRV has its limitations; its changes may be caused not only by emotions but also by other factors. Its indicators can depend on factors such as age, gender, level of physical activity, as well as the presence of illnesses (for example, heart disease), which complicates interpretation. Therefore, for an accurate assessment of the psycho-emotional state, HRV should be combined with other methods, such as psychological tests or clinical observations. Despite this, HRV remains an important tool for studying and monitoring the psycho-emotional state.

Our study found that such a composite index of the psychological state (PS), as proposed by us based on R. McCraty’s method (Mccraty and Shaffer, 2015), the psycho-emotional state index known as the Bayevsky index (Baevskii, 2004), and the regulatory reserves index proposed by us (Chaikovsky et al., 2023), are of great importance in assessing the psychological condition of military personnel. Additionally, parameters of the PS in the time domain, such as SDSD (Standard Deviation of Successive Differences) and PNN50, are also of significant value.

However, it was also found that the amplitude-time parameters of the ECG itself, as well as general composite indices reflecting the state of the myocardium, are of greater significance. Today, it is well known that psychological stress can lead to atrial and ventricular arrhythmias (Lampert, 2015). The influence of acute psychological stress on the duration of the PR interval, QT interval, and QTc interval is also known. The impact of chronic stress on the myocardium, in general, and on the ECG, in particular, has been studied much less. In this regard, our study, which establishes a connection between small changes in the amplitude-time parameters of the ECG and their correlation with a person’s psycho-emotional state, is of significant novelty. We see this as an important additional tool for more reliable objective diagnosis of stress, which has been underestimated previously. Of course, amplitude-temporal changes in the electrocardiogram (ECG) (as well as changes in heart rate variability (HRV) parameters) are not specific for the diagnosis of stress states, as they may also indicate heart diseases, such as heart failure. However, our study included only patients without signs and history of heart diseases. Consequently, the detected changes in the ECG itself clearly indicate the effect of stress on the heart.

Many studies have been conducted to identify the mechanisms by which stress affects the heart. Notably, research on increased cortisol levels, which contribute to hypertension and metabolic changes (Osborne et al., 2020), inflammation and oxidative stress, which can cause myocardial fibrosis and cell apoptosis (Eisenmann et al., 2016), activation of the renin-angiotensin-aldosterone system, which promotes myocardial remodeling (Yalçin et al., 2024), and disturbances in microvascular flow caused by psychological stress (Gebicki et al., 2023). Of course, all these processes influence the shape of the ECG, specifically the amplitude and timing parameters of the ECG, which can be detected through analysis of subtle changes based on artificial intelligence.

Particular emphasis should be placed on the significance of ECG composite indices. It is evident that individual ECG and HRV indicators only reflect partial aspects of the studied phenomenon; moreover, they can be contradictory. Therefore, a comprehensive index is needed that synthesizes the effects of individual components. Consequently, we developed several composite indices, starting with the most integrated, designed to assess the overall functional state of a person, and progressing to more specific indices aimed at evaluating individual important aspects of functional status.

The hardware-software system we developed is a compact, easy-to-use tool, which opens up broad prospects for its application by other independent research groups, and therefore for intensive external validation.

Future research should aim to validate these findings through longitudinal studies. Moreover, further research should lead to the creation of a comprehensive decision rule for predicting the level of stress and the resource-energy component of the functional state of a serviceman, integrated into software that is widely used in military teams.

## 5 Conclusion

The study’s results clearly demonstrate the significant potential for predicting the outcomes of routine psychological assessments for patients based on an in-depth analysis of the electrocardiogram (ECG) and heart rate variability (HRV).

The research established reliable correlations between ECG/HRV parameters and the results of psychological tests. Notably, robust connections were found not only for the indicators of HRV but also for the amplitude-time parameters of the ECG itself, as well as for composite indices that reflect the state of the myocardium.

The highest number of highly reliable connections between the cardiovascular (ECG/HRV) parameters and the psychological tests was found for the Beck Anxiety Scale. This finding highlights the particular sensitivity of these physiological markers of cardiac activity to anxiety levels, which may form the basis for developing objective screening methods.

## Data Availability

The datasets presented in this study can be found in online repositories. The names of the repository/repositories and accession number(s) can be found in this article/supplementary material.
